# Population Genomics of the Fission Yeast *Schizosaccharomyces pombe*

**DOI:** 10.1371/journal.pone.0104241

**Published:** 2014-08-11

**Authors:** Jeffrey A. Fawcett, Tetsushi Iida, Shohei Takuno, Ryuichi P. Sugino, Tomoyuki Kado, Kazuto Kugou, Sachiko Mura, Takehiko Kobayashi, Kunihiro Ohta, Jun-ichi Nakayama, Hideki Innan

**Affiliations:** 1 Graduate University for Advanced Studies, Hayama, Kanagawa, Japan; 2 National Institute of Genetics, Mishima, Japan; 3 Department of Biology, Faculty of Sciences, Kyushu University, Fukuoka, Japan; 4 Department of Life Sciences, The University of Tokyo, Tokyo, Japan; 5 Graduate School of Natural Sciences, Nagoya City University, Nagoya, Japan; University of Cambridge, United Kingdom

## Abstract

The fission yeast *Schizosaccharomyces pombe* has been widely used as a model eukaryote to study a diverse range of biological processes. However, population genetic studies of this species have been limited to date, and we know very little about the evolutionary processes and selective pressures that are shaping its genome. Here, we sequenced the genomes of 32 worldwide *S. pombe* strains and examined the pattern of polymorphisms across their genomes. In addition to introns and untranslated regions (UTRs), intergenic regions also exhibited lower levels of nucleotide diversity than synonymous sites, suggesting that a considerable amount of noncoding DNA is under selective constraint and thus likely to be functional. A number of genomic regions showed a reduction of nucleotide diversity probably caused by selective sweeps. We also identified a region close to the end of chromosome 3 where an extremely high level of divergence was observed between 5 of the 32 strains and the remain 27, possibly due to introgression, strong positive selection, or that region being responsible for reproductive isolation. Our study should serve as an important starting point in using a population genomics approach to further elucidate the biology of this important model organism.

## Introduction

The eukaryotic genome contains several non-coding regions that are crucial for chromosome maintenance and many other basic cellular processes. Obvious examples are centromeres and telomeres, although there are likely to be several other functionally important regions that are yet to be identified [1]–[4]. With an aim to characterize all such functional non-coding elements that lie between the centromere and telomere, which we termed “intermeres”, mainly using model organisms such as the fission yeast *Schizosaccharomyces pombe* and the budding yeast *Saccharomyces cerevisiae*, we recently formed an interdisciplinary consortium funded by MEXT (The Ministry of Education, Culture, Sports, Science and Technology, Japan).

The fission yeast *S. pombe* is a unicellular archiascomycete fungus which has been an excellent model species in molecular and cellular biology, particularly for studying cell-cycle control [5], cytokinesis [6], mitosis and meiosis [7], DNA repair and recombination [3], centromere structure [2], and RNAi-mediated heterochromatin assembly [4]. This is largely because *S. pombe* shares many features with higher eukaryotes that are not present or highly diverged in the budding yeast *S. cerevisiae*. The 12.5 megabase (Mb) genome of the *S. pombe* laboratory strain has already been sequenced [8] and compared with three distantly related fission yeast species [9].

Population genomics is a very powerful approach to study the biology of any organism. For instance, identifying genetic differences among different individuals within a species is crucial in order to understand the genetic basis of phenotypic variation within the species. Also, the genomic pattern of polymorphism provides important clues in identifying functional elements within the genome. Indeed, population genomic surveys have been undertaken in many model organisms, including *S. cerevisiae*, and serve as crucial resources for studying the biology of each organism [10]–[13]. Despite the intensive use of *S. pombe* as a model organism, its genetic variation is poorly understood. To date, only 3 *S. pombe* strains have been sequenced at the whole genome level [9], [ 14], and the only population survey was performed with 5 loci, or 5,777 bp in total [15]. Here, we sequenced the genomes of 32 worldwide *S. pombe* strains. We report the genome-wide pattern of polymorphisms and signatures of selection observed within the genome based on various population genetic analyses. This work should facilitate the discovery of previously unidentified functional elements, in particular functional non-coding elements, within the genome of *S. pombe*.

## Results and Discussion

We sequenced 32 wild strains obtained from the CBS-KNAW Fungal Biodiversity Centre. The sampled strains were chosen to roughly cover the geographic distribution of this species and include 8 from Africa, 5 from Europe, 6 from Asia, 9 from Central and South America, and 4 whose origins are unknown. Sequencing was carried out by using the Illumina MiSeq sequencing platform. We obtained approximately 4 million paired-end reads of 250 base pairs, or 2 Gb per strain (Table 1). After removing low quality reads and bases, the reads were mapped to the *S. pombe* reference genome [8] using Stampy [16] (see Methods for details). On average, >99% of the genome was covered with an average depth of over 70× per sample. Genotype calling was performed using SAMtools [17]. The very high coverage allowed us to adopt a stringent cutoff and obtain 9,375,308 sites (∼75% of the genome) where the genotypes were confidently assigned for all 32 strains, including 99,381 single nucleotide polymorphisms (SNPs). To estimate the error rate, we sequenced 12 regions (Table S1) of ∼450 bp for each of the 32 strains by Sanger sequencing (173 kb sequences in total containing 147 SNPs). No discrepancies were found between the genotype calling and the Sanger sequencing results, suggesting that the SNPs are of very high quality.

**Table 1:** Summary of sequencing.

ID	Strain	Location	Reads ( 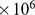 )	Bases (Gb)	Mapped Reads ( 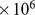 )	Coverage (%)	Average Depth ( 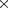 )
1	CBS10391	Unknown	7.38	1.84	4.65	99.0	72.2
2	CBS2628	Pakistan	8.11	2.02	5.73	98.6	91.1
3	CBS1063	Unknown	12.97	3.22	8.79	99.6	133.3
4	CBS2775	Japan	11.02	2.73	7.39	99.6	112.5
5	CBS7335	Spain	11.28	2.80	7.16	99.9	107.7
6	CBS10460	Brazil	5.64	1.40	3.43	99.3	52.4
7	CBS10458	Brazil	4.85	1.21	3.15	99.5	48.0
8	CBS10474	Brazil	6.91	1.69	4.65	99.3	73.1
9	CBS10394	Sri Lanka	5.72	1.42	4.13	99.5	66.9
10	CBS374	Netherlands	12.13	3.01	7.01	99.5	95.0
11	CBS2777	Japan	8.72	2.16	5.22	99.5	83.7
12	CBS1059	Mauritius	11.86	2.95	6.86	99.7	93.5
13	CBS10504	South Africa	8.33	2.07	4.56	99.4	69.2
14	CBS10502	South Africa	6.15	1.53	4.29	99.7	68.3
15	CBS10501	South Africa	4.83	1.20	2.30	99.9	34.9
16	CBS10500	South Africa	12.47	3.10	5.63	99.9	75.0
17	CBS10499	South Africa	6.98	1.74	4.40	99.8	68.7
18	CBS10498	South Africa	6.45	1.60	3.07	99.9	48.0
19	CBS10477	Brazil	6.33	1.57	4.23	99.6	69.0
20	CBS10475	Brazil	5.34	1.32	3.15	99.5	50.8
21	CBS10469	Brazil	6.99	1.73	4.17	99.3	67.3
22	CBS10468	Brazil	7.39	1.83	4.17	99.2	66.6
23	CBS10464	Brazil	5.69	1.41	3.36	99.3	52.0
24	CBS5682	South Africa	8.02	1.99	5.29	99.5	84.6
25	CBS5680	Poland	8.42	2.09	5.45	99.6	86.5
26	CBS5557	Spain	8.87	2.20	5.17	99.8	81.9
27	CBS1061	Unknown	8.57	2.13	4.97	99.6	77.1
28	CBS1058	Indonesia	6.21	1.54	4.18	99.6	66.9
29	CBS1057	Sweden	5.69	1.41	4.12	99.4	66.1
30	CBS1042	Unknown	6.14	1.52	2.85	99.9	44.5
31	CBS357	Jamaica	5.46	1.35	2.93	99.5	45.6
32	CBS352	Indonesia	4.87	1.21	2.72	99.4	42.1
	Average		7.68	1.91	4.66	99.5	71.7

**Figure 1:**
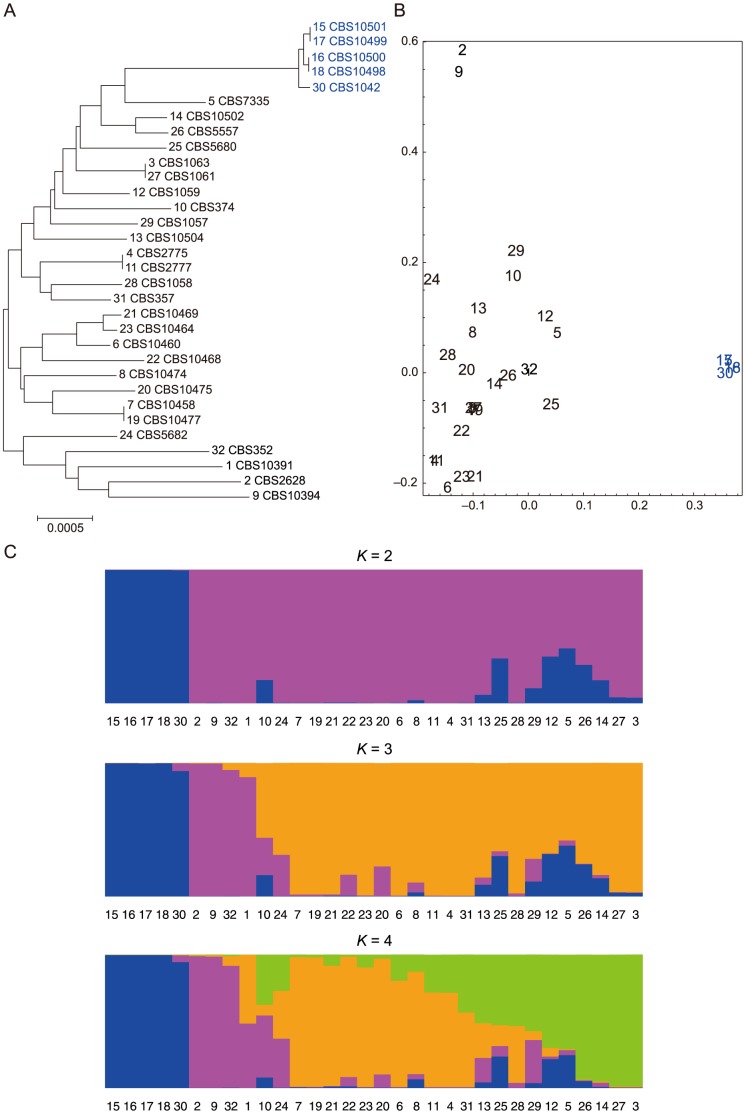
Population structure of *S. pombe*. Neighbor-joining tree (a) and the results of principal component analysis (PCA) (b) and the program STRUCTURE (c). The numbers of the strains correspond to those in Table 1. The 5 samples that strongly cluster together are colored in blue. The results of STRUCTURE with *K = 4* changes when different subsets of randomly chosen SNPs are used, and the result of *K = 4* shown here represents one example. The grouping of strains 15, 16, 17, 18, and 30, and the grouping of strains 2, 9, 32, and 1 are both always observed. The results of *K = 2* and *K = 3* are consistent.

We first examined the population structure of the 32 strains we sequenced. A neighbor-joining (NJ) tree [18] was constructed using all sites without missing data. STRUCTURE [19] and principal component analysis (PCA) were also performed using a subset of SNPs that was randomly selected so that each SNP was separated by approximately 50 kb. The results of the NJ tree, STRUCTURE, and PCA all paint a similar picture (Figure 1). Our samples contain a number of individuals that are highly similar to each other. In particular, 5 strains colored in blue, CBS1042 (#30), CBS10498 (#18), CBS10499 (#17), CBS10500 (#16), and CBS10501 (#15), all show high similarity to each other and are also distinct from the other strains. The reference genome is nearly identical to one of these strains, CBS1042. Apart from these 5 strains, the samples show limited differentiation. The geographic origins of the samples are only weakly reflected.

**Figure 2:**
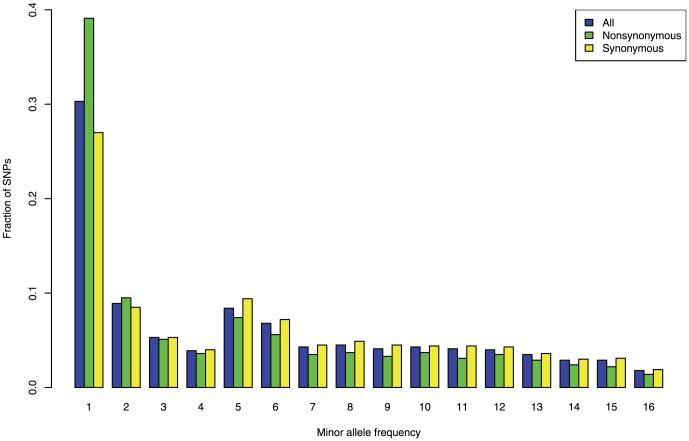
Minor allele frequency spectrum. The frequencies of all sites, nonsynonymous sites, and synonymous sites are shown in blue, green, and yellow, respectively.

Next, we surveyed the pattern of polymorphisms across the genome. The nucleotide diversity 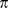 [20] of the whole genome was 0.27% whereas 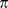 for all synonymous sites was 0.6% (Table 2), indicating that a large part of the *S. pombe* genome is likely to be under selective constraint. The nucleotide diversity was highly reduced at nonsynonymous sites (0.096%), which comprise close to half of the genome, as expected due to stronger selective constraint. Strong purifying selection is also indicated by a much larger number of rare alleles compared to synonymous sites in the minor allele frequency spectra (Figure 2). The slight increase in the frequency spectra around 5 and 6 is most probably due to the 5 strains that are well differentiated as described above. This slight increase indeed disappears when the 5 strains are treated as one sample (Figure S1). Both 5′- and 3′-UTRs (untranslated regions) showed reduced levels of 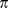 compared to synonymous sites, and also when compared to introns, noncoding RNAs (not including tRNAs, rRNAs, and snoRNAs), and intergenic regions. The nucleotide diversity at intergenic regions was similar to that of introns and noncoding RNAs, and lower than that of synonymous sites. The *S. pombe* genome is relatively compact and the intergenic regions are generally short. The intergenic region is therefore likely to contain unannotated UTRs and promoter regions particularly near coding regions. However, this may not be sufficient to explain the low nucleotide diversity at intergenic regions because the value of 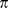 remains similar (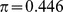) even when 500 bp up- and downstream of the beginning and end of the annotated 5′- and 3′-UTRs are excluded. Thus, the intergenic regions of *S. pombe* are still likely to contain unidentified functional elements that are under selective constraint.

**Table 2:** Summary of nucleotide diversity.

	All	Synonymous	Nonsynonymous	Intron	5′-UTR	3′-UTR	noncoding RNA	Intergenic
All sites	9,375,308	1,285,357	4,332,597	214,537	837,659	1,358,690	54,600	667,464
SNPs	99,381	7,729	4,140	3,582	10,158	15,320	876	11,449
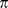	0.271	0.604	0.096	0.440	0.312	0.290	0.434	0.456

The sum of all these categories does not equal the numbers given in “All” as sequences that are too short or contain too many undetermined sites (see Methods) are not included.

**Figure 3:**
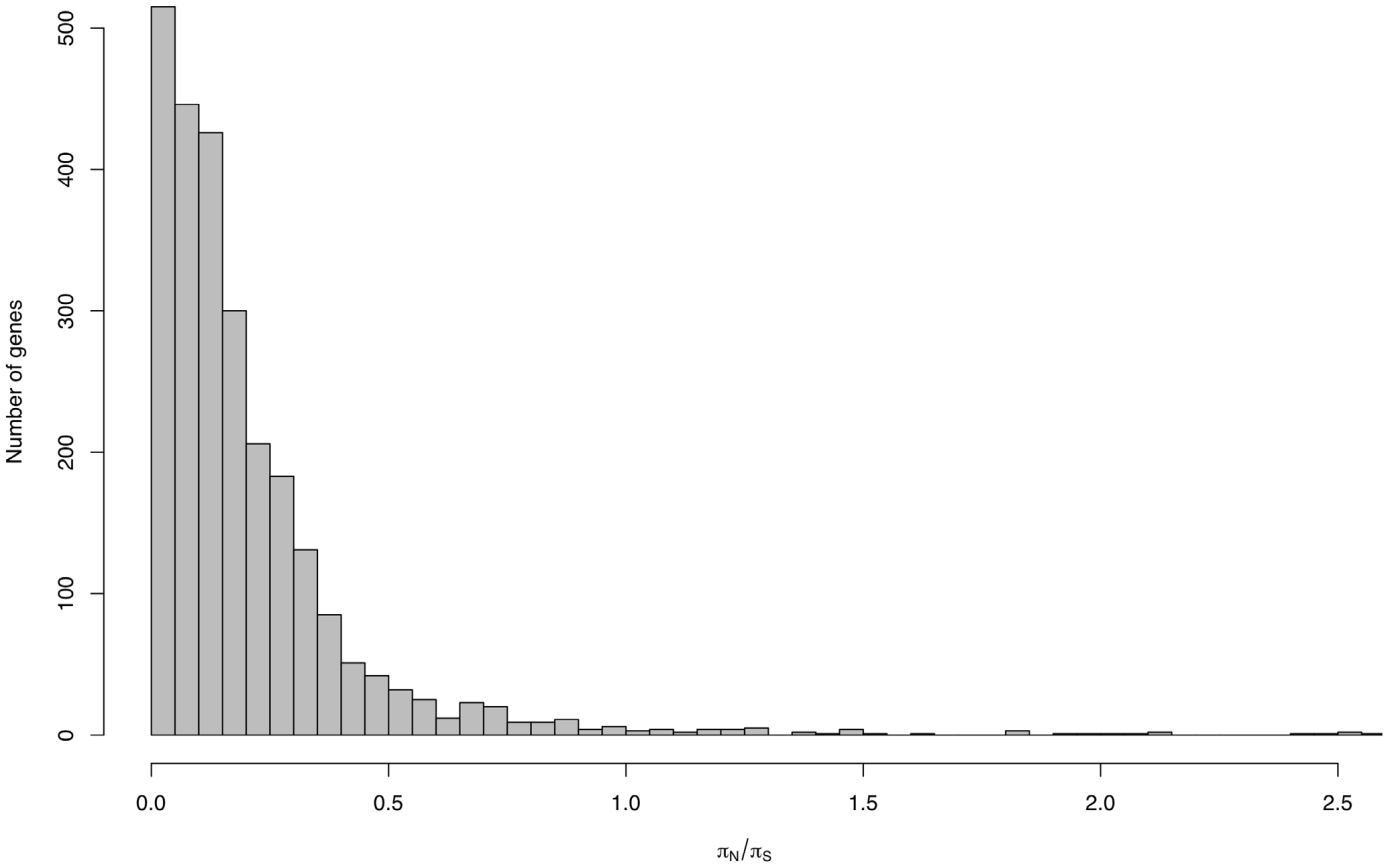
Histogram of 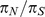. Only genes where more than 200 synonymous sites could be analyzed are included. Four genes with 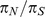 ratios of 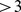 are also not shown.

Selective constraint varies among genes, which should be reflected by the 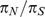 ratio, where 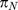 and 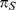 denote the nonsynonymous and synonymous nucleotide diversity, respectively. As expected, the majority of genes show a very low (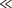
1) 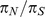 value (Figure 3), suggesting strong selective constraint on these genes, although a very low 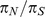 ratio can also arise by chance alone when the numbers of synonymous and nonsynonymous polymorphic sites within a gene are small. A small number of genes exhibited elevated (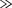
1) 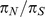 values, which may be due to either relaxation of selective constraint or balancing selection. Balancing selection typically results in the increase of nucleotide diversity and a positive value of Tajima's *D* [21]. The genes with high 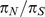 values did not show large values (e.g. >2) of Tajima's *D*, suggesting that relaxed selective constraint is the main reason for the high 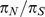. It is possible that some genes under purifying selection would also exhibit high 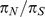 values by chance especially if the number of synonymous and nonsynonymous polymorphic sites are small. We identified a number of genes with high values of Tajima's *D*. A list of genes with the 20 highest Tajima's *D* is provided in Table 3. These genes are candidates for undergoing balancing selection and investigation of these genes may be useful in understanding the basis of phenotypic variation within *S. pombe*. Unfortunately, we could not compute the ratio of nonsynonymous to synonymous substitutions (*dN/dS*) of each gene as the closest species available, *S. octosporus* is too distant that 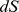 is saturated.

**Table 3:** Top 20 genes with highest Tajima's *D*.

Gene	Position	Tajima's *D*	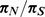	Description
SPAC637.13c	1:4565468–4563609	3.055	0.042	cytoskeletal signaling protein Slm1
SPBC336.05c	2:2748552–2745480	2.925	0.568	small RNA 2′-O-methyltransferase activity
SPAC22A12.06c	1:1165581–1163745	2.916	0.293	serine hydrolase-like
SPAC589.05c	1:3102688–3101103	2.722	0.057	conserved eukaryotic protein
SPBC1718.04	2:3475561–3478365	2.706	0.000	glycerol-3-phosphate O-acyltransferase
SPAC3C7.05c	1:2073591–2071691	2.608	0.063	alpha-1,6- mannanase
SPBC17G9.05	2:2177733–2179449	2.566	0.032	RRM-containing cyclophilin regulating transcription Rct1
SPAC644.14c	1:2700283–2698110	2.552	0.043	RecA family recombinase Rad51/Rhp51
SPAC25G10.05c	1:4305811–4304356	2.548	0.000	ATP phosphoribosyltransferase
SPBC337.03	2:1035538–1036864	2.529	0.188	RNA polymerase II transcription termination factor homolog
SPBC1709.07	2:1110817–1112435	2.442	0.047	3-keto sterol reductase Erg27
SPBC1734.07c	2:1073608–1070188	2.419	0.024	TRAPP complex subunit Trs85
SPCC1322.04	3:1292109–1294029	2.418	0.000	UTP-glucose-1-phosphate uridylyltransferase Fyu1
SPAC14C4.07	1:5240350–5242915	2.366	0.032	membrane transporter
SPCC970.06	3:504777–502927	2.312	0.000	cargo receptor for soluble proteins
SPAC56F8.04c	1:1134356–1133226	2.311	0.044	para-hydroxybenzoate—polyprenyltransferase Ppt1
SPBC337.04	2:1037077–1038385	2.301	0.136	serine/threonine protein kinase Ppk27
SPBP35G2.10	2:981944–986422	2.240	0.315	SHREC complex subunit Mit1
SPBPJ4664.06	2:707511–712426	2.240	0.110	UDP-glucose-glycoprotein glucosyltransferase Gpt1
SPAC30D11.10	1:1101583–1099489	2.227	0.000	DNA recombination protein Rad52

Only genes where more than 200 synonymous sites could be analyzed are shown.

**Figure 4:**
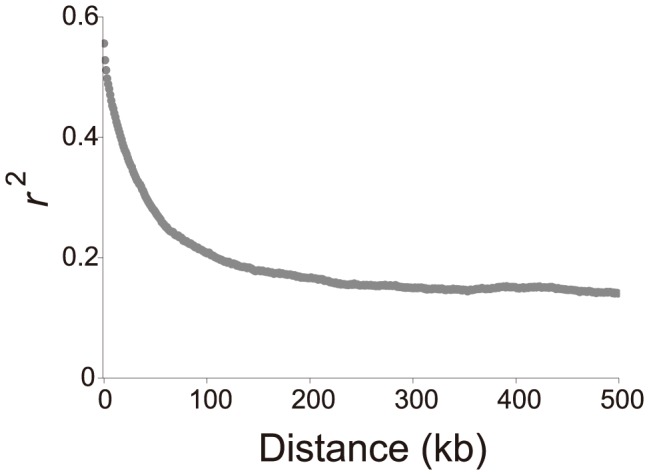
The decay of linkage disequilibrium with distance between SNPs. 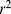
 is plotted as a function of distance in kilobase up to 500 kb. The average 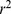 values over each non-overlapping 1 kb bin are shown.

We next examined the spatial distribution of the level of genetic variation, which could reflect the local effect of selection. We first evaluated the decay of linkage disequilibrium against the physical distance using the 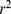 statistic [22]. As shown in Figure 4, 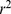 almost reaches a plateau at roughly 100 kb. This pattern of decay indicates that in a neutral situation, it is possible to consider that two regions separated by at least tens of kilobases are almost independent and unlikely to share historical events (i.e., selection). By fitting the observed pattern of decay to the expected pattern of decay [23], the population recombination rate 4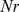, where 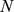 is the effective population size and 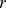 is the recombination rate per base pair per generation, was estimated to be 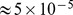. This very low effective rate of recombination (e.g. compared to 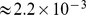 in *S. cerevisiae* [24], and roughly 1/100 of the mutation rate) might be because of the dominant haploid lifecycle of *S. pombe*.

**Figure 5:**
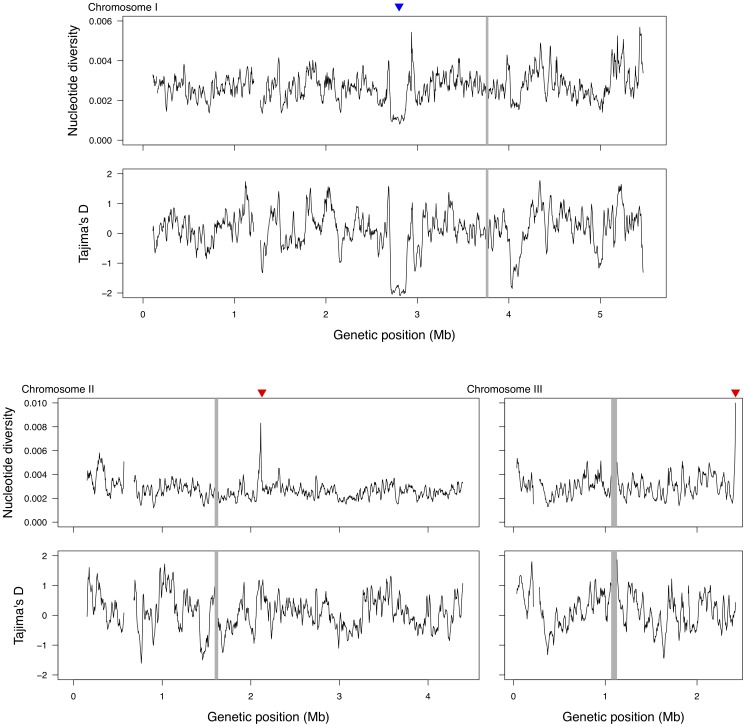
Genome-wide distribution of nucleotide diversity 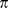 and Tajima's *D*. The values were calculated for each sliding window of 20 kb with an increment of 4 kb. The putative selective sweep region on chromosome 1 with low nucleotide diversity and low Tajima's *D* is indicated by a blue downward triangle. The mating locus region on chromosome 2 and another region on chromosome 3 showing high nucleotide diversity are indicated by red downward triangles. The gray shaded regions correspond to centromeric regions.

We then applied a sliding window analysis of 20 kb with an increment of 4 kb to each chromosome. Provided the estimated degree of linkage, this window size should be reasonable to obtain a rough picture of the large-scale variation of nucleotide diversity across the genome. A large region with reduced polymorphism is a signature of a recent selective sweep, while an exceptionally high level of nucleotide diversity might indicate some other form of selection. As shown in Figure 5, the *S. pombe* genome exhibits little evidence of a selective sweep caused by the recent fixation of an adaptive mutation. One exception is found in the middle of chromosome 1 (around 2.8–2.9 Mb), which displays a long stretch of low nucleotide diversity and negative Tajima's *D*. However, it is interesting to note that this region does contain a certain amount of nucleotide diversity, even though a very recent sweep would typically create a large region with almost no genetic variation. One possibility is that the sweep event was not very recent and that mutations have accumulated after the sweep. On the other hand, careful inspection of this region shows that most of the minor alleles at the polymorphic sites (many of which are singletons) are concentrated in 4 strains, including 3 (CBS10391 (#1), CBS2628 (#2) and CBS 10394 (#9); the fourth one is CBS10468 (#22)) that are amongst the earliest diverging strains according to the NJ tree shown in Figure 1 (see also Table S2). In addition, the remaining strains share an unexpectedly long haplotype (see below). Thus, an alternative possibility would be that the sweep is incomplete and has not yet reached fixation. A list of protein-coding genes in this region is provided in Table S3.

**Figure 6:**
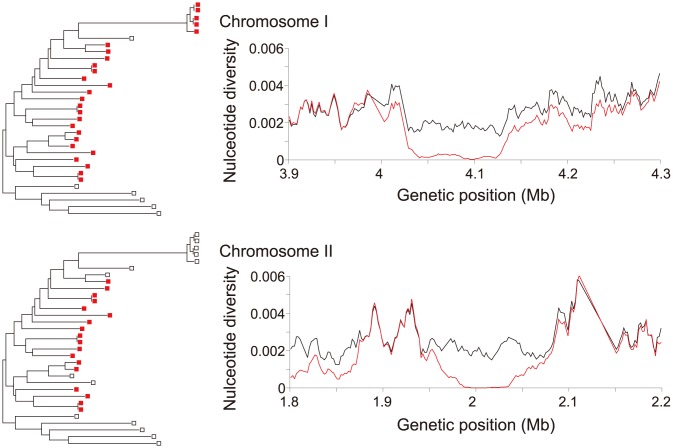
Two examples of putative partial sweep events on chromosomes 1 and 2. The NJ trees shown on the left are the same as in Figure 1a. The strains in red share long haplotypes. Distributions of the nucleotide diversity around the long haplotypes based on a sliding window of 10 kb with a 2 kb increment are shown on the right. Black and red lines represent the nucleotide diversity of all individuals and the nucleotide diversity of the subsamples sharing the haplotypes, respectively.

The signatures of selection may be weak if a sweep has not yet fixed in the species, or if the sweep operates only in a particular geographic region. We explored this possibility by using the pairwise haplotype sharing (PHS) test [25]. This test aims to detect regions where certain individuals share a haplotype over a longer distance than expected, which would indicate a local selective sweep. Indeed, a number of putative partial selective sweep events were identified (see Table S2 for a list of SNPs with the top 0.1% PHS values). Two examples (one on chromosome 1 and the other on chromosome 2) which best illustrate the patterns of polymorphisms expected by partial selective sweeps are shown in Figure 6 (see also Table S4 for lists of protein-coding genes in the two regions). The 2.8–2.9 Mb region of chromosome 1 discussed above also contained several SNPs with high PHS values, although we cannot rule out the possibility that these mutations accumulated in the small number of strains after a complete selective sweep that affected the entire population. In all of the regions showing signatures of partial selective sweeps, the individuals sharing long haplotypes tend to be clustered in the NJ tree, suggesting that in each case the selective sweep was prevented from affecting the entire population. This could be because the sweep started too recently or because differentiation within the population has slowed down the progress of the sweep. Local adaption would be another possible reason for observing a partial sweep.

**Figure 7:**
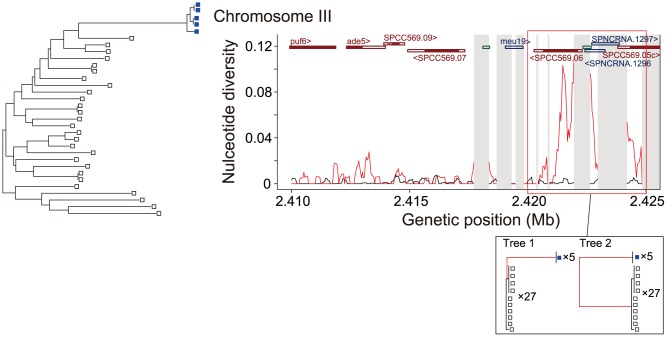
Pattern of SNPs in a region near the end of chromosome 3 showing extremely high nucleotide diversity. The NJ tree shown on the left is the same as in Figure 1a. The 5 strains that are highly diverged from the remaining 27 are indicated by blue. The distribution of the nucleotide divergence between the 5 strains and the remaining 27 (red), as well as the nucleotide diversity within the 27 strains as a control (black) based on a sliding window of 200 bp with 40 bp overlap are shown on the right. Annotated features in the region such as protein-coding genes, noncoding RNAs, and transposable elements are shown above by dark red, dark blue, and green rectangles, respectively. Protein-coding regions within the protein-coding genes are indicated by filled rectangles. The gray shaded regions are those masked in our analysis. The region where the divergence is drastically elevated is boxed in red. Gene trees corresponding to two alternative scenarios that could account for the observed elevated divergence are illustrated below.

Relatively large regions with a high level of nucleotide diversity might be due to some other form of selection. For instance, a region close to the end of chromosome 3 shows the highest level of nucleotide diversity. Closer inspection reveals that this is caused by high differentiation between the 5 strains mentioned above (CBS1042, CBS10498, CBS10499, CBS10500, and CBS10501) and the others (Figure 7). The divergence between these two groups is elevated to as high as >5% at a ∼1 kb region around 2.42 Mb. Although this region is close to the cluster of ribosomal DNA (rDNA) repeats at the end of chromosome 3, it is not clear whether the proximity of this region to the rDNA repeats bears any significance. We note that these rDNA repeats were masked and the high divergence is not an artifact caused by any repetitive sequences. Indeed, this region was included in one of the 12 regions which we verified by Sanger sequencing, and all 31 SNPs called within ∼400 bp were verified. There are two different scenarios that could account for this observed pattern of SNPs in this high diversity region. The first scenario is that a large number of mutations have fixed specifically in the 5 strains (Tree 1 in the box of Figure 7), whereas the second scenario is that the divergence between the two groups at this region is extremely old (Tree 2 in the box). It is unfortunately very difficult to distinguish between these two scenarios especially as there is no adequate outgroup to infer the root of the tree. In the first scenario, we would have to explain why so many substitutions would have occurred only in this specific lineage. One possible explanation might be a mutagenesis effect of recombination considering that the surrounding regions of the rDNA repeats are known as recombination hotspots [26] (although recombination is restricted within the repeats). However, the observed pattern of SNPs instead shows that the two groups have diverged without sufficient shuffling by recombination, suggesting that recombination is reduced (or restricted) in this region. Furthermore, this explanation works only if we assume that the mutagenesis effect is extremely strong specifically in the lineage leading to the 5 strains. As it seems unlikely that the mutation rate in this small region would be suddenly inflated in this specific lineage, we must consider some kind of selection or an unusual demographic event. If selection is the answer, then we must also consider the target of selection. This region does contain a protein-coding gene, SPCC569.06, which is annotated as a membrane protein and has very low (<40%) amino acid similarity to homologs in other *Schizosaccharomyces* species. However, no excess of amino acid substitutions was observed in this gene (19 nonsynonymous and 21 synonymous differences between the two groups). The target of selection could alternatively be non-coding RNA genes or other non-coding regions. In the second scenario where the divergence between the two groups at this region is extremely old, there are a number of different possible explanations. One is that the two groups are reproductively isolated, and that this region is responsible for the isolation. In this case, recombination around this region would be heavily disfavored, resulting in the accumulation of divergence as observed. Another explanation would be introgression from another species, although there is no identified *Schizosaccharomyces* species that is ∼5% diverged from *S. pombe* (the closest identified relative *S. octosporus* has >30% divergence at the amino acid level). Balancing selection might also be a possibility, although in that case it is difficult to imagine that one allele would be present only in the 5 strains that are diverged from the rest also at the genome-wide level. Further characterization is necessary to determine the cause of this unusual observation.

The nucleotide diversity is also high around the mating type region (Figure 5, chromosome 2). *S. pombe* cells alternate between two mating types called P (plus) and M (minus). The mating type is controlled by three homologous cassettes, *mat1*, *mat2-P*, and *mat3-M*, in a ∼30-kb region close to the centromere on chromosome 2. The mating-type determining loci, *mat1*, contains a copy of either *mat2-P* or *mat3-M*, and the mating-type switching occurs as the *mat1* loci is replaced by the other copy by a gene conversion-like process (reviewed in [27]). Although we masked the cassettes and other repetitive sequences in the region (*mat2-P* and the *cenH* element are absent from the reference assembly and thus also not analyzed), we were still able to observe that 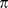 is elevated in this region. Elevated nucleotide diversity around sex determining loci is frequently observed when there is selective pressure to maintain multiple different alleles or haplotypes within the population. However, this is unlikely to apply to the *S. pombe* mating-type region as all individuals should contain both *mat2-P* and *mat3-M*, i.e., the entire genetic information that is required for both mating types. Indeed, despite the high nucleotide diversity, we did not detect any signatures of balancing selection such as a high Tajima's *D*. For this region, the mutagenesis effect of recombination may explain the observation better because 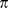 is elevated in this region without any significant increase of Tajima's *D*. The surrounding regions of the mating type region are also known as recombination hotspots [28], and we indeed found an elevated number of detectable recombination events in the SNP pattern (data not shown). A more detailed investigation in the future is required to fully understand the factors influencing the pattern of polymorphisms in such an “unusual” region.

## Conclusions

Here, we have reported the resequencing of the genomes of 32 *S. pombe* strains. Resequencing data and population genomic analyses are extremely valuable for any model organism in order to understand its biology. Indeed, we were able to detect various signatures of selection in the genome of *S. pombe*. For instance, a number of genes showed high Tajima's *D*, which might be subject to balancing selection. In addition, we identified a number of regions showing signatures of selective sweeps. Of particular interest were the two regions with very unusual patterns of SNPs, that any simple scenario of selection cannot explain. At the genome-wide level, we found that polymorphism is reduced in intergenic regions, suggesting strong selective constraint on non-coding regions (i.e., “intermeric” regions as we defined). Our findings should motivate experimental biologists to investigate these genes/regions in more detail. This study should serve as an important starting point for further population genomic analyses of *S. pombe*, which should help enhance our understanding of the biology of this important model organism.

## Methods

### Sequencing, mapping, and genotype calling

Sequencing was performed using the Illumina MiSeq sequencer and MiSeq reagent v2-500 cycle kits (Illumina). The sequencing libraries were prepared from sheared genomic DNA (400–500 bp) with KAPA library preparation kit (Kapa biosystems) and TruSeq-library compatible adaptors (IDT) according to the manufacturer's instruction. Genomic DNA shearing was performed by the AFA system M220 and AFA microTUBE (Covaris) at peak power 140, duty factor 10, cycle/burst 200 at 4°C for 45 seconds. The sheared DNAs were separated by agarose gel electrophoresis and purified by Nucleospin gel clean-up kit (MN) according to the manufacturer's instruction. The resulting short read sequences are available at NCBI under the accession number PRJNA248418. As many of the reads contained several low quality bases, especially towards the 3′-end, a filtering procedure was applied as follows. First, both ends were trimmed until there were at least 10 consecutive bases with a phred quality score of ≥30. Next, 3′-end regions of 150 or 200 bp onwards were removed if either region contained ≥10% of bases with a phred score of <20. After that, reads were removed if the entire read contained ≥10% of bases with a phred score of <20. Adapter sequences reported by fastqc (http://www.bioinformatics.babraham.ac.uk/projects/fastqc/) were trimmed. Reads with <45 bp remaining after the trimming steps were also removed. In addition, reads that consist of a single base, reads containing 3 or more consecutive undetermined bases, pairs where one read is identical to the reverse complement of the other were all removed. If one read of the pair was removed, the other paired-end read was also removed. In order to estimate the error rate of the SNP calling, 12 genomic regions were selected and amplified by PCR using the primers listed in Table S1. The resulting DNA fragments were sequenced using the Sanger sequencing technology using the same primers.

The filtered paired-end reads were mapped to the reference *S. pombe* genome assembly obtained from www.pombase.org using Stampy version 1.0.20 [16]. The default settings were used including the usage of BWA [29] prior to running Stampy. Genotype calling was performed using utilities within the SAMtools package (version 0.1.19) [17]. Potential PCR duplicates were removed (rmdup command), and only reads with a minimum mapping quality of 30 and bases with a minimum base quality of 30 (mpileup -q30 -Q30) were considered. Filtering of the genotype calls was performed as follows using an in-house modified version of the PERL script provided by VCFtools (vcfutils.pl) [30]. A genotype call was retained only if the quality score was ≥30, and if the depth was ≥10 and below 3 times the standard deviation of the average depth of each sample. As *S. pombe* strains are thought to be haploid, heterozygous calls were considered to be erroneous and thus masked. Transposable elements, as well as centromeric and telomeric regions were masked. In addition, >9 mononucleotide repeats, >5 dinucleotide and trinucleotide repeats, together with 10 bp up- and downstream, and also 10 bp up- and downstream of indels were masked. Only sites where the genotypes of all 32 samples could be confidently assigned (i.e., not masked) were retained for further analyses.

### Population genetic analyses

All 9,365,490 sites where the genotype was called for all 32 strains were used for population genetic analyses. The genome-wide nucleotide diversity 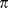 [20] and Tajima's *D* [21] were calculated over 20 kb overlapping sliding windows with an increment of 4 kb. The nucleotide diversity 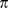 was also calculated separately for protein-coding sequences (CDS), 5′- and 3′-untranslated regions (UTRs), introns, non-coding RNA genes (excluding tRNAs, rRNAs, and snoRNAs), introns, and intergenic regions, based on the annotation from www.pombase.org. Annotated elements which contained ≥1/3 missing sites or whose total length of called sites was <50 bp were removed. For introns, only sites from position 8 to before the branchpoint motif CTAAC (one mismatch allowed) [31] were used. Introns without branchpoint motifs were not included. Intergenic regions were defined as between annotated elements, thus excluding also pseudogenes and repetitive sequences. Excluding 500 bp up- and downstream of the 5′- and 3′-UTRs had little effect on the results. The nucleotide diversity for synonymous and nonsynonymous sites were calculated using the Nei-Gojobori method [32].

A Neighbor-Joining (NJ) tree of the 32 *S. pombe* strains was constructed based on a pairwise *p*-distance matrix that was calculated using all sites without missing data. The population structure was also evaluated using STRUCTURE 2.3 [19]. A randomly selected subset of SNPs that are separated by approximately 50 kb was used. The subset of SNPs was selected as follows. For each chromosome, a first SNP was randomly chosen from the SNPs that were within 50 kb of the 5′-most SNP. Next, the closest SNP to the first SNP out of the SNPs that are at least 50 kb downstream of the first SNP was chosen, and this step was repeated until the 3′-end of each chromosome. A model assuming admixture, correlated allele frequency, and no linkage was used for 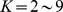 where *K* is the number of populations. The probability of *K* continued to gradually increase as *K* increased. The results were more or less consistent across multiple runs, although the clustering does change when different random subsets of SNPs are used when 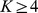. Principal component analysis (PCA) was performed using the same set of SNPs. The eigenvectors were calculated by the procmp function of the R statistical package [33], [ 34].

The decay of linkage disequilibrium (LD) against the physical distance was evaluated by calculating the 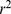 statistic [22] using all sites with a minor allele frequency of ≥10%. The distance between SNPs were divided into non-overlapping bins of 1 kb, and the average 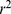 value for each bin was calculated. The population recombination rate 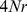, where 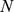 is the effective population size and 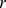 is the recombination rate per base pair per generation, was estimated by fitting the observed pattern of the decay of LD to the expected pattern of decay. The expected pattern was calculated as proposed by [23]. Due to factors such as population structures, 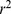 between SNPs that are far apart is likely to deviate from the theoretical expectation which assumes free recombination. Thus, only pairs of SNPs whose distances are between 1 and 100 kb were used to estimate the population recombination rate.

### Pairwise haplotype sharing test

The pairwise haplotype sharing (PHS) test was performed in order to detect footprints of partial selective sweep events [25]. This method calculates the haplotype length shared by a subset of strains, which is normalized against the genetic distance and the degree of genetic similarity among the strains. The genetic (cM) and physical (bp) positions of markers in the *S. pombe* genome were obtained from the NCBI map viewer database [8]. The genetic positions were estimated for all polymorphic sites by fitting a fourth degree polynomial curve to the data on the genetic and physical distances. The PHS statistic as an index of haplotype length was calculated for each allele at a polymorphic site. Suppose that 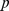 individuals share an allele *A* at position 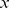. The PHS for the allele *A* is calculated by
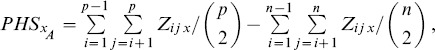
(1)where 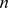 is sample size, and
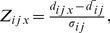
(2)where 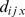 is the genetic distance over which individuals 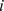 and 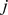 are identical spanning the position 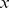, 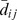 is the genome-wide mean of distances over which the two individuals are identical, and 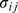 is the standard deviation of the distribution of distances. When calculating 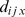, haplotypes were terminated at large clusters of missing data (≥30 kb). Finally, given frequency 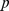, SNPs within the top 0.1% of haplotype length were selected as candidates of partial sweep events.

## Supporting Information

Figure S1 **Minor allele frequency spectrum.** The frequencies of all sites, nonsynonymous sites, and synonymous sites are shown in blue, green, and yellow, respectively. The 5 highly similar strains (CBS1042 (#30), CBS10498 (#18), CBS10499 (#17), CBS10500 (#16), and CBS10501 (#15)) are treated as one sample and sites containing variation within these 5 strains are excluded.
(EPS)

Table S1 **List of primers used for PCR and Sanger sequencing.**
(XLSX)

Table S2 **List of SNPs with significant PHS values.**
(XLSX)

Table S3 **List of protein-coding genes around region of low nucleotide diversity.**
(XLSX)

Table S4 **List of protein-coding genes around regions of putative partial selective sweeps.**
(XLSX)
